# Explosive eruption, flank collapse and megatsunami at Tenerife ca. 170 ka

**DOI:** 10.1038/ncomms15246

**Published:** 2017-05-15

**Authors:** Raphaël Paris, Juan J. Coello Bravo, María E. Martín González, Karim Kelfoun, François Nauret

**Affiliations:** 1 Université Clermont Auvergne, CNRS, IRD, OPGC, Laboratoire Magmas et Volcans, F-63000 Clermont-Ferrand, France; 2 Instituto Volcanológico de Canarias (INVOLCAN), Antiguo Hotel Taoro, Parque Taoro s/n, 38400 Puerto de la Cruz, Spain; 3 Museo de Ciencias Naturales de Tenerife, Calle Fuente Morales, s/n, 38003 Santa Cruz de Tenerife, Spain

## Abstract

Giant mass failures of oceanic shield volcanoes that generate tsunamis potentially represent a high-magnitude but low-frequency hazard, and it is actually difficult to infer the mechanisms and dynamics controlling them. Here we document tsunami deposits at high elevation (up to 132 m) on the north-western slopes of Tenerife, Canary Islands, as a new evidence of megatsunami generated by volcano flank failure. Analyses of the tsunami deposits demonstrate that two main tsunamis impacted the coasts of Tenerife 170** **kyr ago. The first tsunami was generated during the submarine stage of a retrogressive failure of the northern flank of the island, whereas the second one followed the debris avalanche of the subaerial edifice and incorporated pumices from an on-going ignimbrite-forming eruption. Coupling between a massive retrogressive flank failure and a large explosive eruption represents a new type of volcano-tectonic event on oceanic shield volcanoes and a new hazard scenario.

## Abstract (web summary)

Oceanic shield volcanoes flank failures can generate large tsunamis. Here, the authors provide evidence that two tsunamis impacted the coast of Tenerife 170 Ma, the first generated by volcano flank failure and the second following a debris avalanche of the edifice during an on-going ignimbrite-forming eruption.

Massive flank failures of oceanic shield volcanoes are often an order of magnitude larger (tens to hundreds of km^3^) than the largest debris avalanche affecting other types of volcanic edifices such as stratovolcanoes, and represent a potential source of megatsunami^1^,^2^. With a volume of 5 km^3^ the collapse of Ritter Island stratovolcano (Papua New Guinea) in 1888 was the largest historical volcano flank failure and it produced a 10–15 m tsunami on the coasts of the Bismarck Sea^3^. There is an abundant literature on volcanic instability and the possible role of internal versus external factors^1^,^4^,^5^,^6^. Models of evolution of oceanic shields and their rift-zones focus on the relationships between gravitational spreading, the intrusive system and the formation of shallow magma reservoirs^7^,^8^,^9^,^10^,^11^. However, the failure mechanisms and dynamics of flank failures of oceanic shield volcanoes are still poorly documented, thus resulting in great uncertainties on related tsunami hazards^2^,^12^,^13^. Marine conglomerates and megaclasts found at unusually high elevations in Hawaii, Cape Verde, Mauritius and Canary Islands were interpreted as being the result of tsunami waves generated by massive flank failures of oceanic shield volcanoes^14^,^15^,^16^,^17^,^18^,^19^,^20^. These conglomerates are preserved at different elevations, often out of the range of marine highstand deposits. Their composition reflects a mixing of different sediment provenance and assemblages of bioclasts (planktonic, benthic, littoral and subaerial), which are never found in growth position.

Here we document tsunami deposits (marine gravels with pumices) on the north-western flanks of Tenerife, Canary Islands, at altitudes up to 132 m a.s.l. (Fig. 1). The stratigraphy of the tsunami deposits and characterization of pumice clasts found in these deposits allows us to distinguish at least two successive tsunamis, identify their possible source, and demonstrate the association between a massive flank failure (Icod collapse) and an explosive ignimbrite-forming eruption (El Abrigo) both about 170 kyr ago. A unique example of coupled slope instability and explosive activity at an oceanic shield volcano is the relatively small debris avalanche (<200 × 10^6^ m^3^) triggered during the Helecho explosive eruption on the south-eastern flank of Tenerife 733 kyr ago^21^. Apart for this example, such causal links remain speculative, especially for larger-scale flank failures^22^.

## Results

### Characteristics of the tsunami deposits

**Figure 1:**
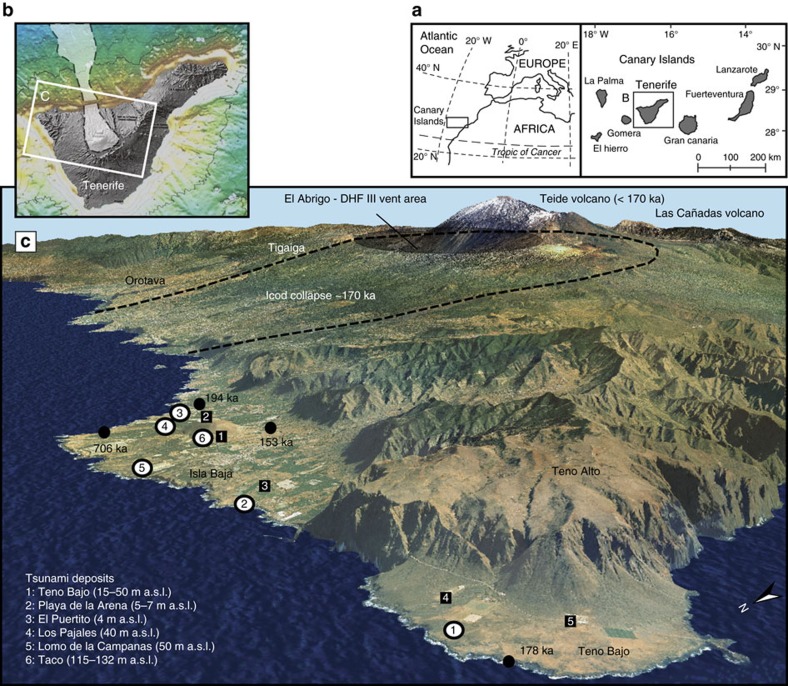
Location and altitude of tsunami deposits on the north-western coast of Tenerife, Canary Islands. (**a**) Location map of the Canary Islands in the Atlantic Ocean; (**b**) Shaded relief view of Tenerife island, showing the Icod debris avalanche on the north flanks of the island; (**c**) 3d view of North-West Tenerife. Tsunami deposits are located on the Isla Baja and Punta de Teno platforms (white dots with numbers). Black squares with numbers correspond to wave gauge used for numerical simulations (Supplementary Table 4). Note limits of the Icod flank failure and Diego Hernandez—El Abrigo caldera in the background. The scar of the failure is filled by lava flows of the Teide volcanic complex.

**Figure 2:**
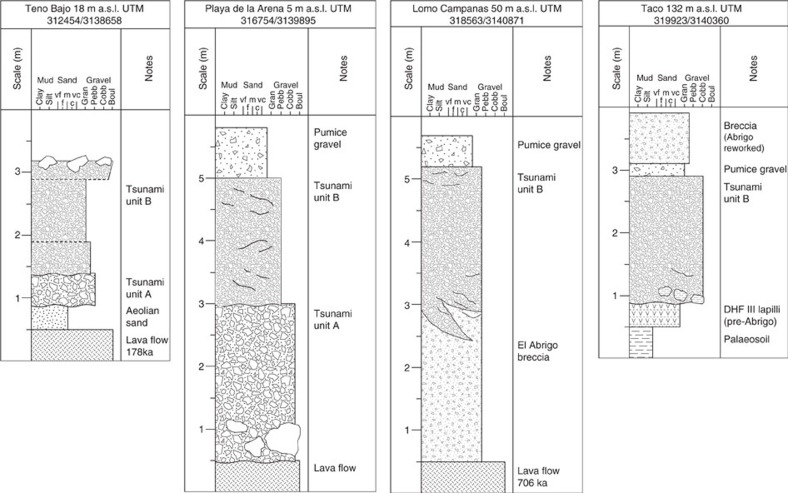
Stratigraphic logs of the main sections. Tsunami unit A is a very coarse-grained gravel that is clast-supported, and poorly sorted (from fine pebbles to boulders). Composition of tsunami unit A is dominated by locally-derived basaltic lavas, but there are also numerous marine bioclasts (bivalve and gastropod shells, foraminiferas, calcareous algae, coral fragments) and rare pumices. Tsunami unit B is a coarse gravel, matrix-supported, and considerably enriched in pumices relative to unit A. Unit B and the underlying El Abrigo breccia have a similar composition: local-derived basalts are mixed with phonolites, hydrothermally altered rocks, syenites, obsidian, pumices, and few marine bioclasts. The contact between tsunami units A,B displays large scour-and-fill features. Unit B is crudely structured into subunits with local variations of grain size and composition. Note that tsunami unit A is absent at elevations higher than 21 m a.s.l. See Fig. 1 for location of the sections.

**Figure 3:**
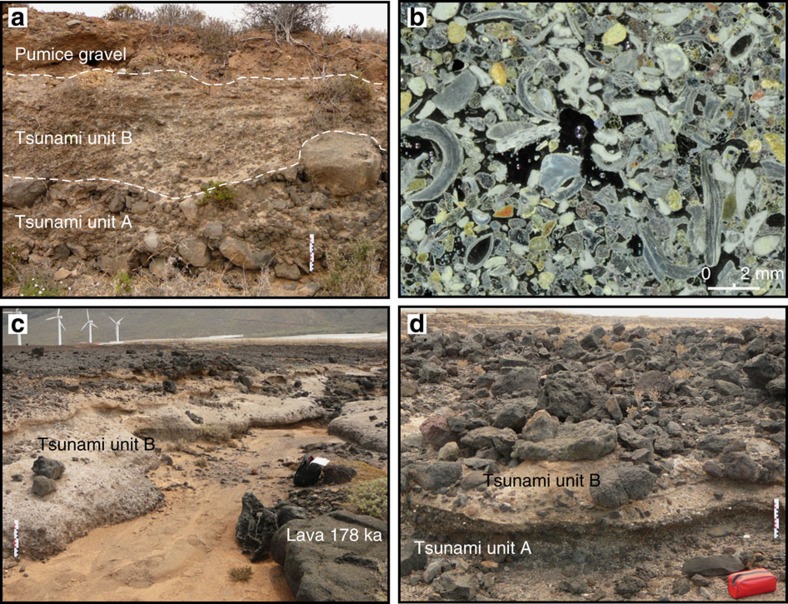
Tsunami deposits at Playa Arena and Teno Bajo. (**a**) Succession of tsunami units A,B at Playa de la Arena (Isla Baja); (**b**) Thin section of tsunami deposits at Playa Arena (tsunami unit A). Note the numerous marine bioclasts (fragments of bivalve shells, bryozoans, coralline algae, and foraminifers); (**c**) General view of the tsunami deposits at Teno Bajo (altitude 18 m a.s.l.) with a lava flow dated 178 ka outcropping on the lower right corner; (**d**) Tsunami unit A eroded by tsunami unit B with floating boulders from the underlying 178 ka lava flow at Teno Bajo. Note the difference between tsunami units A,B in terms of texture, mostly due to the abundance of pumices in unit B. Scale bars in centimeters.

Tsunami deposits are preserved at different locations around Isla Baja and Teno Bajo (Fig. 1). The stratigraphy of the sedimentary sequences slightly differs from one site to another (Fig. 2), due to the nature of the substratum underlying the tsunami deposits, and to the lateral and longitudinal discontinuity of the different tsunami units. At Playa de la Arena, two units of tsunami deposits can be distinguished within a paleo-valley (Fig. 3a). Lower unit A is a massive gravel that is clast-supported, very coarse-grained and poorly sorted (from fine pebbles to 1 m large boulders). The dominant population of clasts comes from basaltic lavas that forms nearby coastal cliffs and platforms, but there are also numerous marine bioclasts (Fig. 3b: bivalve and gastropod shells, foraminiferas, calcareous algae, coral fragments), rare rounded pebbles from the beach, and rare pumices. The thickness of unit A ranges from 40 cm to 1.5 m, but its base is not visible. Upper unit B is also a coarse gravel, but it is slightly finer than unit A, matrix-supported, and considerably enriched in pumices relative to unit A. Unit B is thus a pumiceous gravel, and its composition is much more varied than unit A: local-derived basalts are mixed with phonolites, hydrothermally altered rocks, syenites, obsidian and subangular pumices. The dominant type of pumices is light-green coloured, highly vesiculated, and fibrous. The matrix is sand to silt-sized, and contains abundant bioclasts. The contact between units A and B displays large scour-and-fill features. Unit B is particularly thick (up to 2 m) and crudely structured into subunits with local variations of grain size and composition. The subunits are often separated by a thin layer of very fine material (ash size). There is no vertical size grading in the individual units and subunits, except in some inversely-graded clast-supported lenses. The two units of tsunami deposits are overlain by a consolidated lithic-rich pumiceous gravel that is less than 1 m thick. No marine bioclasts could be found in this gravel, which could correspond to the reworked facies of a lithic-rich ignimbrite (Fig. 3a). Another coastal section of tsunami deposits was found near El Puertito, on the northern coast of Isla Baja (Fig. 1), where tsunami unit A overlies a palaeosoil on a lava flow dated 194 ka (ref. 20).

Tsunami deposits are also exposed on the Teno Bajo peninsula at altitudes between 15 and 50 m a.s.l. (Fig. 3c). Some outcrops have been briefly described^23^,^24^ and a tsunami origin was proposed^25^,^26^. Post-depositional erosion reduced the deposits to small patches on top of a basaltic lava flow dated 178 ka (ref. 27). A 40 cm thick sand layer is intercalated between the lava flow and the tsunami deposits. The stratigraphy of the deposits slightly differs from one section to another, but a synthetic log can be traced as follows (Fig. 2). Lower unit A is a coarse gravel fining landward (from very coarse to medium pebbles, in a very coarse sand matrix), with rare pumice clasts, and particularly rich in fragments of bivalve shells. As in Playa de la Arena, Unit A has no internal structure. The majority of the clasts are angular to subangular fragments of basaltic lavas coming from the underlying lava flows. Unit A is clearly residual here and its thickness ranges between 5 and 50 cm (Fig. 3d). There is no dominant orientation of the elongated clasts. The basal contact between unit A and the sand layer is erosional. Compared to unit A, upper unit B is considerably enriched in pumice clasts, thus forming a 0.5–1.5 m thick pumiceous gravel where different subunits can be distinguished (depending on variations of grain size and proportion of pumices). The indurated matrix is essentially made of fine pumice fragments. Marine bioclasts are also present in various abundances from one subunit to another. Mean grain size ranges from fine to coarse pebbles, but larger clasts up to metric boulders can be found. The lower part of unit B is characterized by a crude stratification forming discontinuous lenses or trains of imbricated and inversely graded clasts. The orientation of the elongated or imbricated clasts is alternatively landward and seaward (Fig. 3d), except in the uppermost subunit where clast imbrication is clearly seaward. It is worth noting that this uppermost subunit is also particularly rich in rounded pumice lapilli and marine bioclasts. Units A and B can be traced respectively up to 320 and 700 m inland (21 and 50 m a.s.l.).

**Figure 4:**
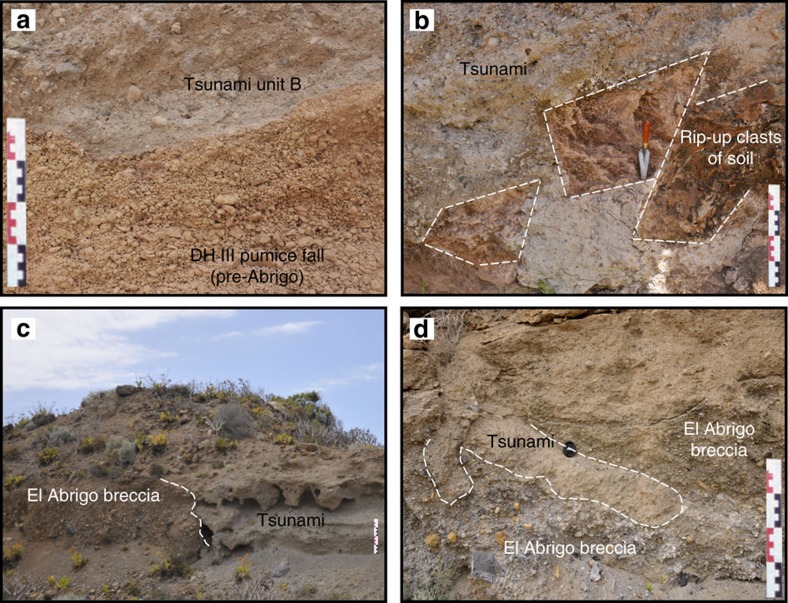
Tsunami deposits at Taco and Lomo de las Campanas. (**a**) Tsunami deposit (pumiceous marine gravel, corresponding to tsunami unit B) at 132 m a.s.l. on the flanks of Taco volcanic cone, eroding pumice fall deposits of the Diego Hernandez III Formation (pre-Abrigo eruption); (**b**) Rip-up clasts of soil as evidence of substrate erosion at the base of tsunami unit B (Taco outcrop); (**c**) Contact between the Abrigo breccia and tsunami deposits (tsunami unit B) at Lomos de las Campanas (50 m a.s.l.). Both units have a similar composition but tsunami unit B is matrix-supported and cemented by carbonates; (**d**) Detailed view of the erosional contact between the Abrigo breccia and tsunami unit B. The contact is characterized by downward injections of the tsunami in the breccia, suggesting basal amalgamation. Scale bars in centimeters.

Traces of the tsunami deposits were identified at several locations around a volcanic cone named Taco and dated 706 ka (ref. 27; Fig. 1). On the western flank of the cone, tsunami unit B was identified at altitudes between 115 and 132 m a.s.l. The main section (quarry at 132 m a.s.l.) displays tsunami unit B as a massive pumiceous gravel eroding a succession of pyroclastic deposits, colluvial deposits and palaeosoils (Fig. 2). The tsunami also truncates a layer of cream-coloured pumice lapilli interpreted as a Plinian fall deposit (Fig. 4a). Thickness of the tsunami gravel is irregular, ranging from 50 cm to 3 m. Erosion of the substratum is illustrated by 20–40 cm large rip-up clasts of the underlying palaeosoil (Fig. 4b). The base of the tsunami deposit is characterized by a 2–5 cm thick fine-grained traction carpet. Composition of the gravel is similar to unit B downslope (Playa de la Arena section): fragments of lava flows of different lithology (from basalts to phonolites), pumices and rare obsidians, and marine bioclasts. Different facies of pumices are present: light green pumices (dominant facies), cream pumices, and banded crystal-rich (feldspars) pumices.

At Lomo de las Campanas (50 m a.s.l.) tsunami unit A is also absent and tsunami unit B directly scours a clast-supported breccia (Fig. 4c,d), which corresponds to the uppermost subunit of the Abrigo ignimbrite (last eruption of the third Diego Hernandez cycle^28^,^29^,^30^,^31^,^32^). The dominant facies of the Abrigo eruption on Isla Baja is a massive lithic-rich ignimbrite, as observed along the coast, but the Abrigo breccia outcrops locally^28^. Tsunami unit B and the Abrigo breccia share the same heterogeneous composition (basaltic and phonolitic lavas, hydrothermally altered lavas, syenites, obsidian, crystal-rich juvenile clasts), but the tsunami gravel is matrix supported and cemented by carbonates. The contact between tsunami unit B and the Abrigo breccia is clearly erosional (Fig. 2), but its interpenetrative geometry (amalgamated contact) suggests that the two events are closely spaced in time, if not simultaneous (Fig. 4d). There is no weathering horizon between the Abrigo breccia and tsunami unit B.

The abundance of bioclasts differs from one site and subunit to another (upper tsunami unit A being richer than lower unit B), and tends to decrease landward. Bioclasts are never in growth position. The terrestrial fauna is represented by rare gastropod shells and two bones of a giant endemic lizard (*Gallotia goliath*). More than 1,000 marine bioclasts were analysed and 123 taxons were determined: 85 gastropods, 31 bivalves, 6 corals and 1 scaphopod (Supplementary Table 1). Bivalve shells such as *G. glycymeris* and *Anadora gibbosa*, and scaphopods *Laevidentalium caudani* are particularly abundant. Biodiversity of the marine fauna is particularly rich and represents a mixing of faunas from different environments (depth, substratum), species of the infra-circalittoral zones being dominant. All taxons can be found nowadays in the Canary Islands. Fragmentation of shells is moderate for the gastropods (25%) and high for the bivalves (46%). Bioturbation affects 28% of the marine bioclasts (incrustation, bioperforation).

### Age of the tsunami

Tsunami deposits are younger than the 178 ka (Teno Bajo, Playa Arena) and 194 ka lavaflows (El Puertito) on which they rest (Fig. 1). The 40 cm thick sand layer intercalated between the tsunami deposits and the 178 ka lava flow at Teno Bajo suggests that the tsunami did not occur immediately after the emplacement of the lava flow. We could not find evidence of tsunami deposits on the 153 ka lava flow of El Palmar volcano^27^, which is very close to Playa Arena (Fig. 1). A chronological link between the tsunami, the last major explosive eruption in Tenerife (El Abrigo^28^,^29^,^30^,^31^,^32^) and a massive collapse of the north flank of the Las Cañadas central edifice (Icod collapse^33^,^34^,^35^) can be established. The age of the uppermost breccia of the Abrigo ignimbrite, which is stratigraphically concomitant with the tsunami, is still controversial. ^40^Ar/^39^Ar ages of feldspars range between 196±6 ka (ref. 29) and 169±1 ka (ref. 36), with discrepancies related to the presence of partly degassed xenocrysts^29^. Nepheline syenites of the breccia were dated at 179±11 ka (ref. 37) (K-Ar), 183±8 ka (ref. 37) (^40^Ar/^39^Ar) and 175±3 ka (K-Ar)^38^. The Icod submarine debris avalanche has been dated ∼170 ka from shallow seismic^33^. The age of the Icod turbidite in the Agadir Basin was estimated at 155–175 ka (ref. 39) (coccolithophore biostratigraphy) and 165±15 ka (ref. 40) (Oxygen Isotope Stages). This is concordant with the 161±5 ka (ref. 38) and 158±5 ka (ref. 41) ages of the oldest post-collapse lavas filling the Icod embayment onshore (collected in two different water galleries below the Teide volcanic complex). Thus, published ages point to a major suite of large-magnitude events (explosive eruption, flank collapse and tsunami) affecting Tenerife *ca.* 170–175 kyr ago. Note that the present-day altitudes of the different tsunami outcrops are out of the range of documented MIS 5.5 marine highstands in the Canary Islands (<12 m a.s.l.)^42^, except for the Playa de la Arena and El Puertito outcrops.

### Origin of the pumices

**Figure 5:**
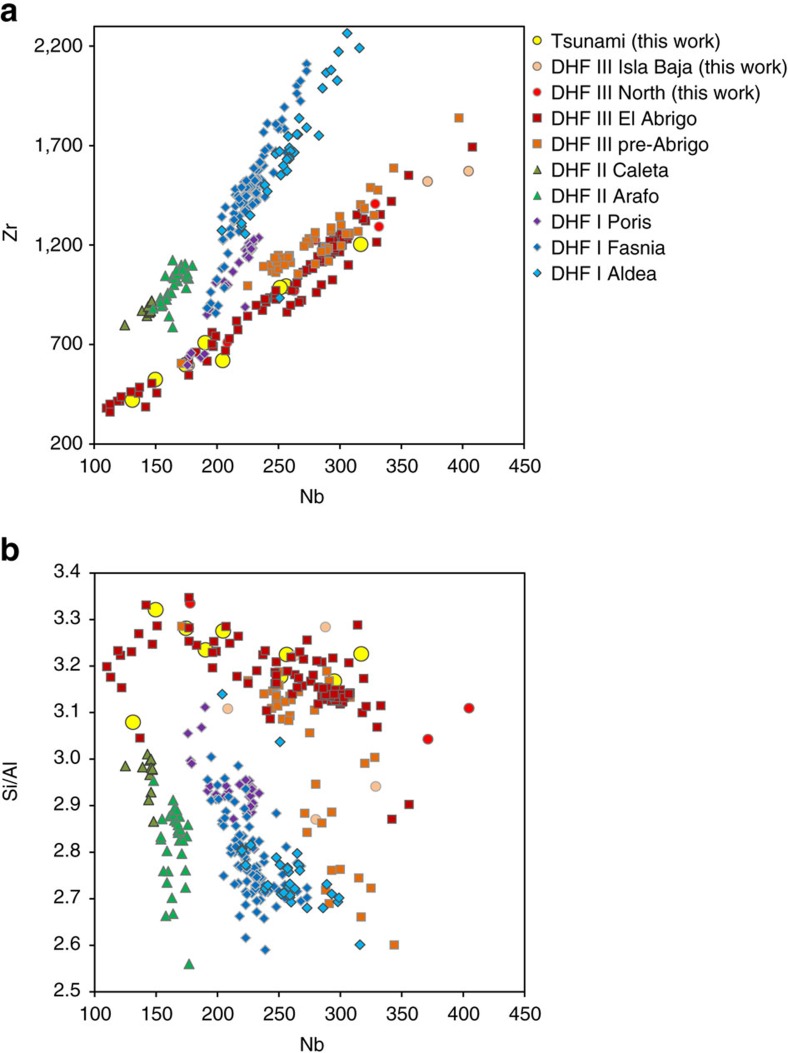
Pumice geochemistry. Nb versus Zr (**a**) and Nb versus Si/Al (**b**) of pumices collected in the Tenerife tsunami deposits, compared to pumices of the Diego Hernandez Formation^43^,^44^. Error bars (2σ for major elements and ±3% for trace elements) are within the size of the symbols.

The pumice clasts found in the tsunami deposits resemble the great variety of pumices of the third Diego Hernandez Formation (DHF III), in terms of colour (light green, grey, cream pumices), crystallinity (from near-aphyric pumice to crystal mush), and texture (fibrous, coarsely to finely banded). The phenocryst assemblage is similar to the DHF phonolites (alkali feldspars, clinopyroxene, biotite, magnetite, titanite). Major and trace elements analyses (and especially the Si/Al and Nb/Zr ratios) are consistent with a DHF III origin of the pumices incorporated in the tsunami deposits (Fig. 5). Despite small differences between the DHF phonolitic units in terms of mineralogy and geochemistry, Si/Al and Nb/Zr values allow us to distinguish two lineages^43^,^44^. DHF III products have significantly higher Si/Al and higher Nb/Zr ratios than DHF I and DHF II (Fig. 3). Complex history of titanite fractionation, melting of pre-existing syenite plutons, and mingling with mafic magma explains these variations in trace elements within the different DHF units^43^,^44^.

Light green and cream pumice collected in the tsunami deposits both have a typical DHF III signature (Fig. 5). The green pumice is tentatively interpreted as the northern counterpart of plinian fall deposits identified at the base of the Abrigo ignimbrite in the south of the island^28^. Both deposits share the same composition, colour and texture (high-Nb/Zr, fibrous, near-aphyric, light green pumice). The occurrence of a plinian fall phase at the onset of the Abrigo eruption remains controversial because fallout deposits are absent (or not preserved) below the ignimbrite for most of the outcrops^28^,^30^,^31^. The cream pumice in the tsunami deposits comes from a Plinian fall deposit that is clearly eroded by the tsunami on the western flank of Taco (Fig. 3a: pumice lapilli). The upper part of the cream pumice fall deposit displays traces of pedogenesis suggesting that it is not synchronous with the tsunami and Abrigo eruption (it was thus possibly deposited by a DHF III pre-Abrigo eruption such as Benijos^29^). The pumice gravel overlying tsunami unit B at Playa de la Arena, Lomo Campanas and Taco (Fig. 2) represents a pumice-rich reworked facies of the Abrigo breccia.

## Discussion

High-Nb/Zr phonolitic magmas of Tenerife are often correlated with large caldera-forming eruptions leading to the destruction of the shallow magma reservoirs, such as the Granadilla^36^,^45^ and El Abrigo eruptions^43^,^44^. The Abrigo eruption was triggered by input of fresh mafic magma into a crystal-rich, water-undersaturated phonolitic—syenitic reservoir^46^. The roof of this reservoir was at 130±50 MPa (*ca.* 4–5 km below the surface or 1–2 km below the ocean surface). The Abrigo eruption has an estimated volume of 20 km^3^ (with only 1.8 km^3^ now exposed onshore^29^), a maximum thickness of 20 m in the Bandas del Sur area (southern flanks of the Las Cañadas volcano), 3–5 m in the Orotava valley and Tigaiga massif (North), and 2–3 m in Isla Baja (Northwest). The uppermost vent-derived lithic breccia of the Abrigo ignimbrite, which is associated with tsunami unit B in Isla Baja, is interpreted to record the onset of caldera collapse^31^. Up to 40% of the lithic clasts come from the pre-existing hydrothermal complex. The eruption led to a near-complete evisceration of the shallow magma system^46^. Note that the interpretation of the Las Cañadas caldera walls (flank failure versus caldera subsidence) and occurrence of a caldera collapse *s.s.* during the El Abrigo eruption is beyond the scope of this paper.

However, we demonstrate that there is converging evidence that the Abrigo eruption and the Icod flank collapse are linked. In the proximal field the Icod submarine debris flow deposits are up to 20 km wide and 105 km long, with an estimated volume of 110±40 km^3^ (refs 34, 35). Different depositional lobes can be distinguished, suggesting a multistage collapse^34^,^47^. The Icod debris flow is correlated to a large turbidite (210±20 km^3^) in the Agadir and Madeira abyssal plains at distances up to 1,000 km away from Tenerife^48^,^49^. The Icod turbidite consists of a series of seven vertically stacked volcaniclastic subunits separated by erosive discontinuities and/or short mud intervals^49^. The lower three subunits have larger volumes (80–90 km^3^), palagonitized glass and higher biogenic carbonate content (20–50% in the lowermost subunit), compared to the upper four subunits (∼20 km^3^ each). There is a vertical trend in the composition of volcanic glass, from mafic-intermediate lavas in the lower subunits to phonolitic-trachytic glass in the upper subunits. Glasses of El Abrigo—DH III composition appears in the last subunit only. The Icod event was thus a multistage retrogressive failure, affecting successively the submarine flanks of the island and the basaltic shield, and then the phonolitic-trachytic series of the Las Cañadas edifice^49^. The onset of the first Icod failures pre-dates the Abrigo eruption, which is only recorded in the last turbidite subunit.

On the northern submarine flank of Tenerife, the Icod flank collapse and Abrigo eruption are thus recorded by three proximal lobes of debris flow, seven distal turbidite subunits, and two tsunami units onshore. The spatial distribution of facies and characteristics of newly discovered tsunami deposits in Tenerife allows us to propose a complete scenario of flank collapse, major explosive eruption and subsequent tsunamis 170 kyr ago. The event started with successive submarine-to-subaerial flank failures—large enough to generate tsunami waves—inundate Isla Baja and Teno Bajo, and leave a massive gravel rich in marine bioclasts at altitudes up to ∼20 m a.s.l. (tsunami units A). This first stage detached more than 100 km^3^ from the north flank of Tenerife, including the submarine flanks, and might have triggered a significant decompression of the magma reservoir. The northwestern part of Tenerife was mantled by pumice fall deposits and lithic-rich ignimbrite before the second tsunami occurred and incorporated large amount of pumices (tsunami units B). This second pumice-rich tsunami reached higher altitudes (deposits at 132 m a.s.l. in Taco and 50 m a.s.l. in Teno Bajo) than the first one, and it was generated at an advanced stage of the eruption, that is, at a late and exclusively subaerial stage of the Icod failures^49^. The inferred source is a debris avalanche of the Las Cañadas edifice at the end of the eruption, which generated a larger tsunami on the coasts of Tenerife (tsunami units B), compared to the previous submarine failures (tsunami units A).

**Figure 6:**
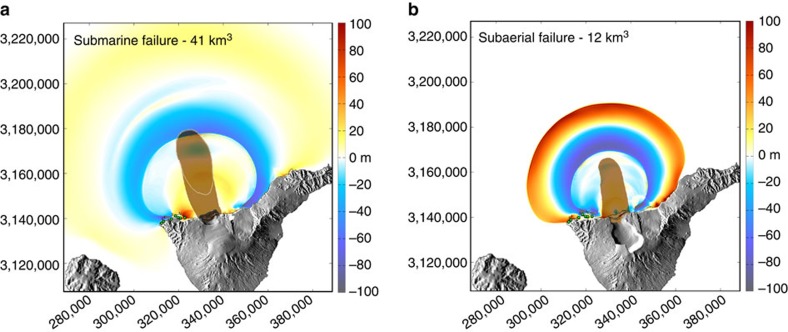
Numerical simulations of failures on the northern flanks of Tenerife and related tsunami. Snapshots after 500 s of simulation. Submarine failure (**a**) and subaerial failure (**b**), respectively, correspond to scenarios 1b and 3b in Supplementary Table 4.

Thus, the volume of the successive turbidites and flank failures is not the only parameter controlling the amplitude of tsunamis impacting the coasts of Tenerife. Indeed, the characteristics of tsunamis generated by flank failure do not depend only on the volume of the sliding mass, but also on its origin (subaerial, submerged or both), dynamics (for example, initial acceleration, maximum velocity, retrogressive behaviour, deformation, single block or granular), and water depth (the height of the leading wave being limited by water depth in open ocean)^50^,^51^. Numerical simulations of failures on the northern flank of Tenerife (Supplementary Table 4) confirm that a 12–15 km^3^ en masse failure of the subaerial flank of the Las Cañadas edifice generate tsunami waves high enough to submerge the cone of Taco (Fig. 6), where tsunami deposits (unit B) were preserved at an altitude of 132 m. Whatever the scenario and rheology of the failure, the Teno Bajo peninsula is flooded until altitudes higher than 50 m a.s.l. (that is, higher than the tsunami deposits preserved).

Marine conglomerates at high altitudes (up to 132 m a.s.l.) on the northwestern flanks of Tenerife are thus an evidence of tsunami generated by the Icod flank failures *ca*. 170 ka. The stratigraphy and composition of the deposits suggest at least two successive tsunamis, the second one reworking pumices from the DHF III pre-Abrigo and Abrigo explosive eruptions. The proposed scenario agrees with the turbiditic record of the events and demonstrates that the retrogressive flank failure and explosive eruption are linked. The first submarine-to-subaerial failures were tsunamigenic (tsunami units A) and might have participated to the triggering of the eruption. The last subaerial failure is associated to the largest tsunami (tsunami units B) and final destruction of the Las Cañadas edifice. This is the first evidence of coupled large explosive eruption (ignimbrite-forming) and massive flank failure at an oceanic shield volcano. Further investigations might reveal similar associations in Tenerife (link between the Roques de Garcia debris avalanche^35^ and previous explosive phases of Las Cañadas volcano?) or the Cape Verde Islands (Santo Antão^52^, Fogo^53^). The scenario inferred from this study has major implication in terms of hazard assessment since the tsunamis extends the impact of the collapse and eruption to a regional scale. Considering the high density of population and economic infrastructures along the coasts, such a scenario would have a devastating impact. The monitoring and warning systems are unsuited to dealing with such events, but this study represents an important step towards an integrated volcano-tsunami risk evaluation.

## Methods

### Taphonomic and geochemical analyses

For taphonomic analysis of the bioclasts found in the tsunami deposits (Supplementary Table 1) we followed the methodology used by Yesares-Garcia and Aguirre (2004)^54^. Major element compositions (Supplementary Tables 2 and 3) were analysed by ICP-AES (Jobin-Yvon ULTIMA C) at LMV (Laboratoire Magmas and Volcans, Clermont-Ferrand France) after XRF dissolution. For trace elements analysis (Supplementary Tables 2 and 3), samples were dissolved in a HNO_3_–HF mixture, heated for 24 h and then evaporated. After dissolution, fluoride precipitates were dissolved with several cycles of additions of 7N HNO_3_ and 6N HCl and evaporations. Whole-rock trace elements were obtained by solution Inductively-Coupled Plasma Mass-Spectrometry (ICP-MS, Agilent7500, Agilent Technologies) at LMV. A standard-sample bracketing was used: the BIR-1standard was measured every two samples and the sample measurements where normalized by linear interpolation to the Geo-ReM (http://georem.mpch-mainz.gwdg.de/) preferred values of the BIR-1.

### Numerical modelling

Numerical simulations of debris avalanches and tsunamis were realized using Volcflow numerical package, which is able to dynamically model any kind of mass flow and related tsunami generation, propagation and inundation inland^12^,^18^,^55^. Solving for the non-linear shallow water equations on a regular Cartesian grid is employed using an Eulerian explicit upwind numerical scheme. Open boundaries are prescribed at the limits of the computational domain. Second order processes such as wave breaking near the shore and physical dispersion are neglected. Parameters of the different scenarios of flank failure simulated are summarized in Supplementary Table 4.

### Data availability

All data generated or analysed during this study are included in this published article (and its Supplementary Information files) or available from the corresponding author on reasonable request.

## Additional information

**How to cite this article:** Paris, R. *et al*. Explosive eruption, flank collapse and megatsunami at Tenerife ca. 170 ka. *Nat. Commun.* **8,** 15246 doi: 10.1038/ncomms15246 (2017).

**Publisher's note**: Springer Nature remains neutral with regard to jurisdictional claims in published maps and institutional affiliations.

## Supplementary Material

Supplementary Information Supplementary Tables.
