# Supplementary material for: Explosive eruption, flank collapse and megatsunami at Tenerife ca. 170 ka
Source: Nat Commun. 2017 May 15;8:15246. doi: 10.1038/ncomms15246 (PMC5440666; doi:10.1038/ncomms15246)
Supplement: Supplementary Information — Supplementary Tables. [file ncomms15246-s1.pdf]

	Bathymetric zonation					Substrate	
	S	M	I	C	B	S	R
CNIDARIA							
Cladocora debilis Milne Edwards & Haime, 1849				•			•
Caryophyllia smithii Stokes & Broderip, 1828				•	•		•
Sphenotrochus andrewianus Milne Edwards & Haime, 1848				•	•		
Lophelia pertusa (Linnaeus, 1758)					•		
Asterosmilia prolifera (Pourtalès, 1871)				•	•		•
Balanophyllia cf. *thalassae* Zibrowius, 1980					•		•
GASTROPODA							
Patella aspera Lamarck, 1819		•					•
Patella crenata d´Orbigny, 1840		•					•
Patella piperata Gould, 1846		•					•
Tectura virginea (O.F. Müller, 1776)		•				•	
Diodora gibberula (Lamarck, 1822)		•					•
Diodora graeca (Linnaeus, 1758)		•					•
Emarginula fissura (Linnaeus, 1758)			•	•			•
Haliotis tuberculata coccinea (Reeve, 1846)		•	•				•
Clanculus berthelotii (d´Orbigny, 1840)		•					•
Jujubinus exasperatus (Pennant, 1777)			•			•	
Jujubinus cf. *gravinae* (Dautzenberg, 1881)			•			•	
Gibbula magus (Linnaeus, 1758)			•			•	
Calliostoma cf. *lithocolletum* Dautzenberg, 1925			•	•	•	•	•
Danilia tinei (Calcara, 1839)					•		
Bolma rugosa (Linnaeus, 1767)			•	•			•
Tricolia pullus (Linnaeus, 1758)				•			
Cerithium lividulum Risso, 1826			•	•			•
Bittium latreilli (Payraudeau, 1826)			•	•			•
Bittium incile Watson, 1897							
Turritella turbona Monterosato, 1877			•	•		•	
Monophorus thiriotae Bouchet, 1985				•		•	
Cerithiopsis sp.							
Eumetula bouvieri (Dautzenberg & Fischer, 1896)			•	•	•	•	
Tectarius striatus (King & Broderip, 1832)	•	•					•
Rissoa lia (Monterosato, 1884)			•			•	
Rissoa sp.							
Alvania cancellata (Da Costa, 1779)			•	•			•
Crisilla sp.							
Zebina paivensis (Watson, 1873)			•			•	
Vermetus triquetrus Bivona-Bernardi, 1832		•					•
Capulus ungaricus (Linnaeus, 1758)				•	•		•
Trivia monacha (da Costa, 1778)		•	•				•
Erato voluta (Montagu, 1803)			•	•		•	•
Natica sp.							
Polinices lacteus (Guilding, 1834)			•			•	
Phalium granulatum (Born, 1778)			•	•		•	
Cymatium corrugatum (Lamarck, 1816)			•			•	
Cymatium tranquebaricum (Lamarck, 1816)			•			•	
Bufonaria marginata (Gmelin, 1791)			•			•	
Bursa scrobilator (Linnaeus, 1758)			•	•			•
Stramonita haemastoma (Linnaeus, 1767)		•	•				•
Coralliophilla sp.							
Marginella glabella (Linnaeus, 1758)		•	•			•	
Volvarina olivaeformis (Kiener, 1834)		•				•	
Granulina guancha (d´Orbigny, 1840)		•				•	
Gibberula oryza (Lamarck, 1822)		•				•	
Mitra sp.			•			•	
Vexillum zebrinum (d´Orbigny, 1840)		•					•
Chauvetia crassior (Odhner, 1932)			•			•	
Colubraria reticulata (Blainville, 1829)			•			•	
Gemophos viverratus (Kiener, 1834)		•					•
Tritia denticulatus (A. Adams, 1852)			•	•		•	
Tritia incrassatus (Ström, 1768)		•	•			•	
Tritia reticulatus (Linnaeus, 1758)			•	•		•	
Columbella adansoni Menke, 1853		•	•				•
Mitrella broderipi (Sowerby, 1844)			•	•		•	
Mitrella pallaryi (Dautzenberg, 1927)			•	•		•	
Fusinus hernandezi Hadorn & Rolán, 2009			•			•	
Bivetiella similis (Sowerby, 1833)			•			•	
Mitromorpha swinneni Midsuf, 2011			•			•	
Bela nebula (Montagu, 1803)							

Mangelia multilineolata (Deshayes, 1835)			•			•	
Raphitoma sp.							
Discotectonica discus (Philippi, 1844)			•			•	
Heliacus fallaciosus (Tiberi, 1872)			•			•	
Philippia hybrida (Linnaeus, 1758)			•			•	
Psilaxis krebsi (Mörch, 1875)			•	•		•	
Retusa tornata (Watson, 1866)			•			•	
Ringicula conformis Monterosato, 1882			•			•	
Bulla mabillei Locard, 1897		•				•	
Trimusculus mammillaris (Linnaeus, 1758)	•	•					•
SCAPHOPODA							
Laevidentalium caudani (Locard, 1896)			•			•	
BIVALVIA							
Arca noae Linnaeus, 1758		•	•	•	•		•
Barbatia barbata (Linnaeus, 1758)			•	•		•	
Anadara gibbosa (Reeve, 1844)			•	•		•	
Striarca lactea (Linnaeus, 1758)		•	•	•	•		•
Glycymeris glycymeris (Linnaeus, 1758)			•	•	•	•	
Limopsis aurita (Brocchi, 1814)			•	•	•	•	
Pecten maximus (Linnaeus, 1758)			•	•		•	
Aequipecten opercularis (Linnaeus, 1758)			•	•	•	•	
Flexopecten flexuosus (Poli, 1795)			•	•	•	•	
Bractechlamys corallinoides (D´Orbigny, 1839)			•	•	•	•	•
Spondylus senegalensis Schreibers, 1793			•				•
Anomia ephippium Linnaeus, 1758			•	•			•
Lima lima (Linnaeus, 1758)			•	•	•		•
Ostrea edulis Linnaeus, 1758			•	•			•
Chama gryphoides Linnaeus, 1758			•	•		•	•
Cardita calyculata (Linnaeus, 1758)			•	•	•		•
Astarte fusca (Poli, 1795)			•	•		•	
Acanthocardia aculeata (Linnaeus, 1758)			•	•		•	
Laevicardium oblongum (Gmelin, 1791)			•	•		•	
Ervilia castanea (Montagu, 1803)			•	•		•	
Venus casina Linnaeus, 1758			•	•		•	
Globivenus effossa (Philippi, 1836)			•	•	•	•	•
Timoclea ovata (Pennant, 1777)			•	•	•	•	
Gouldia minima (Montagu, 1803)			•	•	•	•	
Callista chione (Linneaus, 1758)			•	•		•	

Supplementary Table 1 - Habitat of the marine bioclasts found in the Teno tsunami deposits (Tenerife, Canary Islands). (S) supralittoral; (M) mesolittoral; (I) infralittoral; (C) circalittoral; (B) bathyal. Sustrate: (S) fine sediments (muds, sands); (D) rocky.

Pumices in tsunami deposits

Sample	TE 2	TE 2 bis	TE 2 ter	T05-04-14-8	T05-04-14-8'	T05-04-14-6	T05-04-14-1	T05-04-14-7	T05-04-14-7'
	Punta Teno					Isla Baja (Taco)			
SiO_2	54.97	53.91	55.01	60.01	55.44	60.58	58	59.89	59.08
Al_2O_3	16.78	16.43	17.87	18.95	16.7	18.78	17.93	18.57	18.6
Fe_2O_3	3.4	3.53	6.11	3.58	3.05	3.58	3.44	3.52	4.16
MgO	3.12	4.49	3.04	0.46	1.94	0.55	1.1	0.65	1.12
CaO	7.74	7.73	4.67	1	8.82	0.87	5.07	2.02	2.41
Na_2O	7.67	7.67	6.68	9.38	7.84	9.19	8.13	8.63	8.07
K_2O	5.1	4.79	3.96	5.59	5.1	5.38	5.09	5.6	5.16
TiO_2	0.85	0.92	1.76	0.71	0.8	0.73	0.86	0.79	0.97
MnO	0.17	0.17	0.18	0.24	0.16	0.23	0.18	0.21	0.22
P_2O_5	0.16	0.24	0.46	0.09	0.16	0.11	0.2	0.12	0.2
Total %	*99.97*	*99.87*	*99.73*	*100*	*100*	*100*	*100*	*100*	*100*
Cs	0.64	1.02	0.9	2.12	0.9	2.14	1.21	1.70	1.77
Rb	77.12	86.26	66.85	165.33	87	176.18	107.04	143.21	140.75
Ba	157.92	927.42	1993.62	21.06	210.24	40.62	647.03	103.78	326.20
Th	10.31	14.12	9.68	30.48	12.81	28.71	17.03	24.73	24.91
U	3.91	3.69	2.42	7.63	3.24	7.16	4.26	6.24	6.11
Nb	204.71	174.58	131.26	295.11	149.87	317.11	190.37	256.41	251.47
Ta	12.88	9.52	7.21	15.97	9.57	15.85	10.86	14.88	13.99
La	99.2	95.36	88.32	147.5	93.63	157.83	110.07	136.44	133.28
Ce	197.23	172.04	168.87	252.93	179.7	266.35	202.00	245.82	235.93
Pr	21	17.98	18.97	23.83	19.16	24.30	20.60	24.37	23.10
Pb	8.23	7.45	6.26	14.63	7.17	17.07	9.05	12.26	12.70
Sr	147.51	280.62	641.75	17.17	158.33	23.05	151.87	65.08	179.16
Nd	69.28	59.08	68.74	70.89	64.86	73.15	66.96	76.46	72.31
Zr	618.66	597.1	419.39	1 211.91	523.71	1 202.80	708.56	992.63	985.21
Hf	12.29	11.81	8.6	23.87	10.63	23.17	13.85	19.55	19.43
Sm	10.51	9.01	11.37	10.5	10.13	10.85	10.41	11.47	10.97
Eu	1.83	2.18	3.77	1.68	2.11	1.82	2.36	2.10	2.18
Gd	1.16	1.09	1.25	8.38	7.66	8.69	7.96	9.08	8.74
Tb	7.63	7	8.83	1.32	1.11	1.39	1.17	1.36	1.31
Dy	6.28	5.93	6.64	8.2	6.17	8.36	6.79	8.07	7.67
Y	25.55	33.64	34.37	49.82	30.89	52.82	36.15	46.10	43.09
Ho	1.16	1.16	1.22	1.67	1.16	1.65	1.29	1.59	1.50
Er	2.98	3.05	3.07	4.99	3.17	5.03	3.63	4.55	4.38
Yb	2.48	2.91	2.62	5.12	2.78	5.07	3.37	4.47	4.36
Lu	0.32	0.4	0.36	0.75	0.39	0.74	0.48	0.64	0.62

Supplementary Table 2 - Major and trace elements composition of pumice clasts in the tsunami deposits (ICP-AES and ICP-MS). Major element values recalculated to volatile-free oxide totals of 100 wt.%. Trace elements in ppm.

Diego Hernandez Formation (DHF) III pumices

	DHF III Isla Baja			DHF III Orotava		DHF III Tigaiga (North flank)			
Sample	TE 12	T05-04-14-5	T05-04-14-9	T06-04-14-9	T06-04-14-10	T07-04-14-1	T07-04-14-11	T07-04-14-12	T07-04-14-14
SiO_2	55.6	60.23	60.03	61.61	60.32	60.17	64.51	55.62	61.48
Al_2O_3	16.68	19.79	19.31	18.76	20.51	19.36	20.02	30.4	21.42
Fe_2O_3	3.83	4.16	3.77	3.34	3.97	5.54	3.48	5.94	3.7
MgO	4.17	1.73	1.25	0.75	0.68	2.23	0.7	0.64	0.43
CaO	7.68	0.76	1.08	0.64	0.67	2.32	1.01	1.2	0.73
Na_2O	6.1	7.83	8.28	8.22	8.11	5.04	5.21	2.61	5.94
K_2O	4.44	4.54	5.25	5.72	4.73	3.54	4.05	1.83	5.31
TiO_2	0.94	0.59	0.53	0.6	0.68	1.38	0.74	1.23	0.67
MnO	0.17	0.26	0.31	0.22	0.22	0.15	0.17	0.05	0.24
P_2O_5	0.26	0.1	0.2	0.13	0.11	0.27	0.11	0.46	0.08
Total %	*99.87*	*100.01*	*100*	*100*	*100*	*100*	*100*	*100*	*100*
Cs	1.18	2.71	2.69	2.13	2.75	1.50	1.59	0.36	1.98
Rb	84.96	184.11	200.78	180.59	196.53	105.56	120.07	22.51	168.35
Ba	736.85	77.36	18.85	16.40	44.86	1 217.65	87.18	730.50	78.01
Th	15.52	40.81	43.24	27.82	36.11	18.40	23.41	33.11	33.58
U	3.52	8.18	9.37	7.17	9.13	3.13	4.72	2.51	7.34
Nb	177.81	371.47	404.83	287.98	328.87	208.47	262.98	331.90	280.09
Ta	8.92	14.76	16.44	13.86	14.12	10.75	13.80	18.45	14.16
La	101.22	194.43	184.93	139.79	184.76	197.18	124.09	66.42	131.37
Ce	170.80	267.43	276.41	224.94	242.96	261.13	216.51	141.65	229.67
Pr	17.32	23.98	22.46	19.39	24.32	37.66	20.05	11.11	19.09
Pb	8.26	20.44	21.79	17.12	20.39	11.18	14.52	14.76	16.34
Sr	412.06	43.08	39.00	14.12	43.91	538.36	229.92	393.03	90.43
Nd	54.94	66.17	60.38	55.82	67.08	118.78	61.70	35.29	54.77
Zr	594.94	1 519.29	1 571.25	1 145.79	1 407.06	710.01	969.12	1 292.09	1 223.78
Hf	11.66	29.08	29.50	21.75	25.74	13.39	18.33	24.88	23.61
Sm	8.22	9.35	8.46	7.93	9.41	19.21	9.25	5.62	7.80
Eu	2.14	1.62	1.42	1.30	1.51	4.92	1.74	1.45	1.28
Gd	0.97	8.15	7.33	6.43	7.55	15.59	7.19	4.58	6.34
Tb	6.41	1.35	1.27	1.06	1.23	2.26	1.13	0.74	1.00
Dy	5.44	8.59	8.24	6.41	7.58	12.74	6.56	4.54	6.26
Y	31.72	63.44	59.99	39.75	51.16	74.06	37.41	23.80	37.42
Ho	1.09	1.82	1.76	1.32	1.55	2.39	1.27	0.91	1.31
Er	2.89	5.93	5.74	4.09	4.94	6.77	3.84	2.72	4.01
Yb	2.83	6.37	6.27	4.34	5.32	6.05	3.70	2.73	4.42
Lu	0.40	0.95	0.93	0.63	0.78	0.85	0.54	0.39	0.66

Supplementary Table 3 - Major and trace elements composition of pumice clasts of the Diego Hernandez Formation III in North Tenerife (ICP-AES and ICP-MS). Major element values recalculated to volatile-free oxide totals of 100 wt.%. Trace elements in ppm.

	FLANK FAILURE CHARACTERISTICS									TSUNAMI FLOW DEPTH				
Scenario	rheology			volume	area		runout	thickness		wave gauge				
	frictional	*plastic*	*en masse*		*land*	*sea*				***1***	***2***	***3***	***4***	***5***
				km^3	km^2	km^2	km	m		m	m	m	m	m
**1a**		x		41	622	33	37	511		1	0	80	61	40
**1b**		x	x	41	639	31	45	373		2	0	65	32	18
**2a**		x		15	203	67	29	412		1	0	53	26	7
**2b**		x	x	15	372	75	45	782		60	16	128	72	59
**2c**	x		x	15	201	50	28	538		0	0	0	0	0
**2d**	x			15	304	44	44	637		0	0	10	0	0
**3a**		x		12	234	168	39	258		1	0	45	24	4
**3b**		x	x	12	283	224	46	363		18	2	88	60	35

Supplementary Table 4 - Parameters of the different scenarios of flank failure used for numerical simulations. Scenario 1 is a 41 km^3 submarine failure corresponding to the lower turbidite subunits in the Agadir and Madeira abyssal plains[49]. Different rheologies are tested : Mohr-Coulomb frictional law (with ϕ_{bed} = 2° ; angle of dynamic friction between the sliding mass and the ground surface), constant retarding stress (with T = 50kPa ; retarding stress). Scenarios 2 and 3 are 12-15 km^3 subaerial failures, scenario 3 being located at higher altitudes on the flanks of the edifice. The maximum thickness is measured at the shoreline. See figure 1 for location of the wave gauges.
